# Supplementary material for: An Operational DNA Strand Displacement Encryption Approach
Source: Nanomaterials (Basel). 2022 Mar 6;12(5):877. doi: 10.3390/nano12050877 (PMC8912636; doi:10.3390/nano12050877)
Supplement: Supplementary file 1 [file nanomaterials-12-00877-s001.zip › nanomaterials-1576413-supplementary.pdf]

nanomaterials

MDPI

Supplementary Materials

An Operational DNA Strand Displacement Encryption Approach

Enqiang Zhu [1], Xianhang Luo [1], Chanjuan Liu [2,*] and Congzhou Chen [3]

[1] Institute of Computing Science and Technology, Guangzhou University, Guangzhou 510006, China; zhuenqiang@gzhu.edu.cn (E.Z.); 2112006164@e.gzhu.edu.cn (X.L.)
[2] School of Computer Science and Technology, Dalian University of Technology, Dalian 116024, China
[3] School of Electronics Engineering and Computer Science, Peking University, Beijing 100871, China; chencongzhou@pku.edu.cn
* Correspondence: chanjuanliu@dlut.edu.cn

Supplementary Materials:

Table S1. DNA coding.

	Group 1 Code-G		Group 2 Code-TT		Group 3 Code-TA	
**No.**	**Character**	**DNA codon**	**Character**	**DNA codon**	**Character**	**DNA codon**
1	Space	AT	n	AT	.	AT
2	e	CT	s	CT	u	CT
3	shift	TC	h	TC	,	TC
4	t	TG	r	TG	w	TG
5	a	AC	d	AC	m	AC
6	o	AG	l	AG	f	AG
7	i	CG	c	CG	y	CG
8	g	AAT	3	AAT	;	AAT
9	p	AAC	4	AAC	q	AAC
10	b	AAG	5	AAG	z	AAG
11	v	CAT	6	CAT	<	CAT
12	-	CAA	7	CAA	=	CAA
13	(	CAC	8	CAC	%	CAC
14	)	CAG	9	CAG	+	CAG
15	k	CCA	j	CCA	*	CCA
16	0	CCT	x	CCT	?	CCT
17	1	CCG	/	CCG	>	CCG
18	2	CCC	:	CCC	tab	CCC
19	return	AAAT	$	AAAT	{	AAAT
20	^	AAAA	&	AAAA	}	AAAA
21	_	AAAC	~	AAAC	“	AAAC
22	#	AAAGC	[	AAAGC	\	AAAGC
23	@	AAAGT	]	AAAGT	|	AAAGT
24	!	GTCGCCG				
25	Page break	GTCTACCC				

Table S2. DNA encoding and decoding rules.

Rule	1	2	3	4	5	6	7	8
00	A	A	T	T	G	G	C	C
01	G	C	G	C	A	T	A	T
10	C	G	C	G	T	A	T	A
11	T	T	A	A	C	C	G	G

Table S3. Synthetic DNA complexes.

	**DR-module**
B	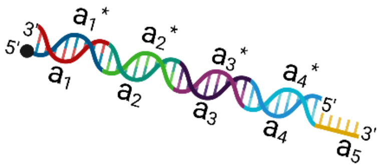
D	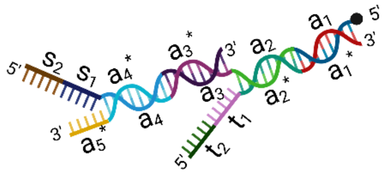
G	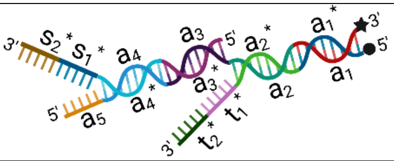
	**CR-module**
B	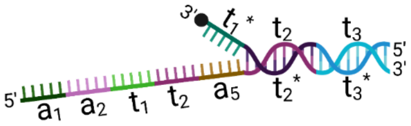
D	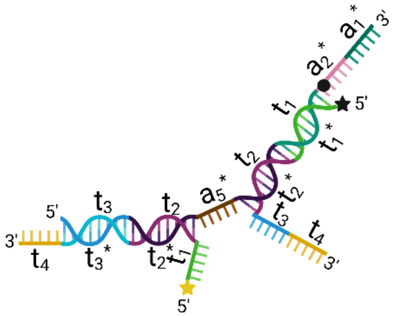

Table S4. DNA sequence design.

NO.	Name	Sequences(5′-3′)
**DNA sequence design of DR-module**		
Note: the yellow bases are toehold domains, the blue bases are marked with BHQ1 quencher, and the red bases are marked with TET fluorophore.		
B-d/ D-d	$a_1^*a_2^*a_3^*a_4^*a_5^*$	GAGTAATTTGTGTGTGGAGATGTGGTAGAGTGGAGTATTAGGAGTTTGGAAGGATTGAG-TGGTGGAGTA
B-u	$a_1a_2a_3a_4$	CACCACTCAATCCTTCCAAACTCCTAATACTCCACTCTAC-CACATCTCCACACACAAATTACTC
A	$a_1a_2a_3a_4a_5$	TACTCCACCACTCAATCCTTCCAAACTCCTAATACTCCACTCTACCACATCTC CACACACAAATTACTC
D-u1	$s_2s_1a_4a_4$	TTAGGAGATGCACCACTCAATCCTTCCAAACTCCTAATACTC
D-u2	$t_2t_1a_2a_1$	AGGGTGGTTACACTCTACCACATCTC CACACACAAATTACTC
G-u1	$a_3^*a_4^*s_1^*s_2^*$	GAGTATTAGGAGTTTGGAAGGATTGAGTGGTGCATCT CCTAA
G-u2	$a_1^*a_2^*t_1^*t_2^*$	GAGTAATTTGTGTGTGGAGATGTGGTAGAGTGTAACCACCCT
DNA sequence design of CR-module		
NO.	Name	Sequences(5′-3′)
B-d	$a_1a_2t_1t_2a_5t_2t_3$	CCTAACATCTTACTCCACTCTACCACATCTCCAAACTCCTAATACTCCACTCTAC-CACATCTCCACACACAAATTACTC
B-u	$t_1^*t_2^*t_3^*$	GAGTAATTTGTGTGTGGAGATGTGGTAGAGTGGAGTA
A/ D-u1/ D-u2	$t_1t_2t_3t_4$	TACTCCACTCTACCACATCTCCACACACAAATTACTCCACCACTCAATCCTTC
D-d	$a_1^*a_2^*t_1^*t_2^*a_5^*t_2^*t_3^*$	GAGTAATTTGTGT GTGGAGATGTGGTAGAGTGGAGTATTAGGAGTTTGGAGATGTGG-TAGAGTGGAGTA AGATG TTAGG

Table S5. DNA XOR operation.

**XOR**	**A**	**G**	**C**	**T**
**A**	A	G	C	T
**G**	G	A	T	C
**C**	C	C	A	G
**T**	T	T	G	A

Table S6. DNA ADD operation.

**ADD**	**A**	**G**	**C**	**T**
**A**	A	G	C	T
**G**	G	A	T	C
**C**	C	T	G	A
**T**	T	C	A	G

Table S7. Example of groupCS.

To illustrate the method, consider the following example, where the example 1 illustrates the case that the length of D_1 meet the requirements after the first *k*-round shift, while the example 2 illustrates the case that the length of D_1 does not meet the requirements after the first k-round shift.		
Example 1		
1.	Let *D_0=AGTTACCGCG*, i.e., l_0=10, and we would like to extend *D_0* to a new DNA sequence D_1 with length l', $l' \geq l =$ 20.	
2.	Transform D_0 into (0,1)-sequence S according to Table S2, S=00011110110110001101. Then, we set $k = 2\lceil l/l_0 \rceil = 4$ and perform k rounds of cyclic shift; see the following.	
	Initial	S =00011111001010011001
	Round 1	S =00111110010100110010<br>Q=00111110010100110010
	Round 2	S =11111001010011001000<br>Q=0011111001010011001011111001010011001000
	Round 3	S =11110010100110010001<br>Q=001111100101001100101111100101001100100011110010100110010001
	Round 4	S=11001010011001000111<br>Q=00111110010100110010111110010100110010001111001010011001000111001010011001000111
3.	Now, divide Q into $\left\lceil \frac{80}{8} \right\rceil = 10$ groups, denoted by Q_1=00111110, Q_2=01010011, Q_3=00101111, Q_4= 10010100, Q_5=11001000, Q_6= 11110010, Q_7=10001101, Q_8=00011100, Q_9=10100110, Q_{10}=01000111. Then, delete Q_2,Q_3,Q_6,Q_7 and Q_{10}, and transform the other groups into DNA sequence D_1=ATTGTAAGGCTCAGTAAATA.	
4.	The requirements $l' \geq l$ is satisfied, and output D_1.	
Example 2		
1.	Let *D_0=AGTCTGCATG*, i.e., l_0=10, and we would like to extend *D_0* to a new DNA sequence D_1 with length l', $l' \geq l =$ 20.	
2.	Transform D_0 into (0,1)-sequence S according to Table S2, S=00011110110110001101. Then, we set $k = 2\lceil l/l_0 \rceil = 4$ and perform k rounds of cyclic shift; see the following.	
	Initial	S =00011110110110001101
	Round 1	S =00111101101100011010<br>Q=00111101101100011010
	Round 2	S =11110110110001101000<br>Q=0011110110110001101011110110110001101000
	Round 3	S =11101101100011010001<br>Q=001111011011000110101111011011000110100011101101100011010001
	Round 4	S=10110110001101000111<br>Q=00111101101100011010111101101100011010001110110110001101000110110110001101000111
3.	Now, divide Q into $\left\lceil \frac{80}{8} \right\rceil = 10$ groups, denoted by Q_1=00111101,Q_2=10110001, Q_3=10101111, Q_4= 01101100, Q_5=01101000, Q_6= 11101101, Q_7=10001101, Q_8=00011011, Q_9=01100011, Q_{10}=01000111. Then, delete Q_1,Q_3,Q_6,Q_7,Q_8,Q_9 and Q_{10}, and transform the other groups into DNA sequence D_1=ACGTCGATCGGT.	
4.	The length of D_1 is 12, l'=12 < l, which does not meet the requirements. So, add *k* rounds of cyclic shift. $k = 2\left\lceil \frac{l-l'}{l_0} \right\rceil = 2$. See the following.	
	Initial	S =10110110001101000111
	Round 1	S =01101100011010001111<br>Q=01101100011010001111
	Round 2	S =10110001101000111101<br>Q=0110110001101000111110110001101000111101
5.	Now, divide Q into $\left\lceil \frac{40}{8} \right\rceil = 5$ groups, denoted by Q_1=01101100, Q_2=01101000, Q_3=11111011, Q_4= 00011010, Q_5=00111101. Then, delete Q_3, Q_4 and Q_5, and transform the other groups into DNA sequence and splice them after D_1. D_1 was extended to ACGTCGATCGGTCGATCGGT. Now the length of D_1 is 20, l'=20 = l.	
6.	The requirements $l' \geq l$ is satisfied, and output D_1.	

Table S8. An example of the algorithm BioEN.

1.	Encrypted string abc. Transform abc into a DNA sequence D_1 according to the tri-phase transformation. D_1=GCG-GATTTTAA.
2.	Set 2-11-2 as the seed for the initial key. Transform it into DNA sequence according to the rules listed in Supplementary Table S1, and then extend it to a new DNA sequence D_2 with length at least that of D_1 according to groupCS. D_2=GAACGCCCGCCC.
3.	Divide D_2 into three groups, where group2-1=GAAC, group2-2=GCCC, group2-3=GCCC. According to the middle two digits of the 01 sequence of 8 bits corresponding to each group, label group 1 by *add*, group 2 by *xor*, and group 3 by *xor*.
4.	Divide D_1 into three groups, where group1-1=GCGG, group1-2=ATTT, group1-3=TAA.
5.	Condute *add* operation between group1-1 and group2-1; Condute *xor* operation between group1-2 and group2-2; and condute *xor* operation between group1-3 and group2-3. The result D_3=ACGTGGGGTCC.
6.	Then, we obtain the ciphertext C=' U ë STX by transforming D_3 into ASC II code by Supplementary table S2.

Table S9. Proof of Key Space Analysis.

1.	Obtain the key 2-11-2 through experiments. Transform the key into DNA sequence X according to the rules listed in Table S1. X=GCCCGCAAGCCGGCCGGCAAGCCC. Transform X into (0,1)-sequence S according to the rules in the first column of Table S2. S= 011010100110000001101001011010010110000001101010. Use $R^i(S)$ to represent the generated (0,1)-sequence after the ith round of shift. i starts from 1, and i+1 after shift one round. $R^0(S) = S$.	
2.	If $i \equiv 1\ (mod\ 2)$, then $R^i(S)$ moves the first place to the last place on the basis of $R^{i-1}(S)$; if $i \equiv 0\ (mod\ 2)$, then $R^i(S)$ moves the first two digits to the last two digits on the basis of $R^{i-1}(S)$.	
	$R^1(S)$	110101001100000011010010110100101100000011010100
	$R^2(S)$	010100110000001101001011010010110000001101010011
	$R^3(S)$	101001100000011010010110100101100000011010100110
	…	…
	$R^{32}(S)$	011010100110000001101001011010010110000001101010
	$R^{33}(S)$	110101001100000011010010110100101100000011010100
	$R^{34}(S)$	010100110000001101001011010010110000001101010011
	…	…
3.	After 32 shifts, the generated 01 sequence is repeated, i.e., $R^{32+j}(S) = R^j(S)$, where j is a nonnegative positive integer. The length of the (0,1)-sequence generated by 32 rounds of shift determines the size of our key space. Each round of shift can produce a 48-bit (0,1)-sequence, and 32 rounds can produce 1536 bit (0,1)-sequence. Therefore, key space is 2^{1536}.	

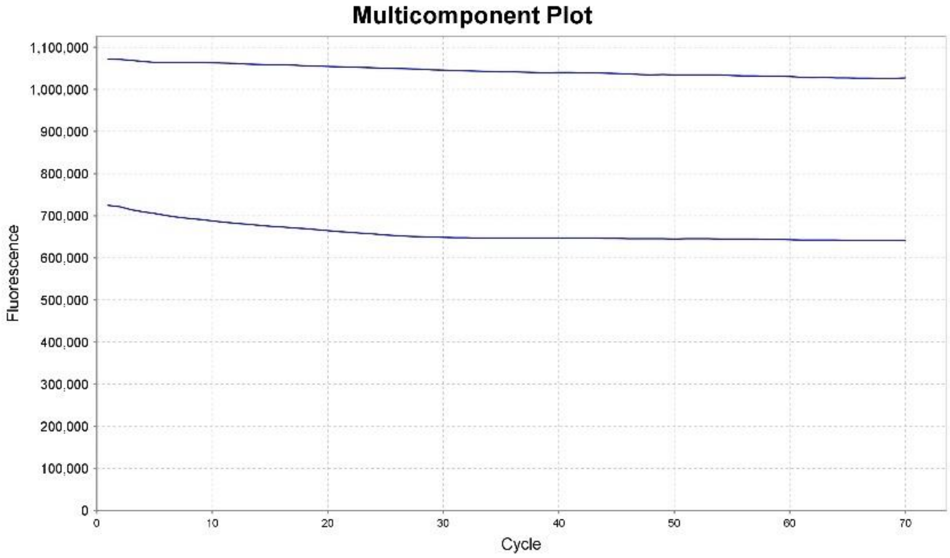

Figure S1. The fluorescence intensity changes when the concentration ratio of ABDG is 1:1:1:1.

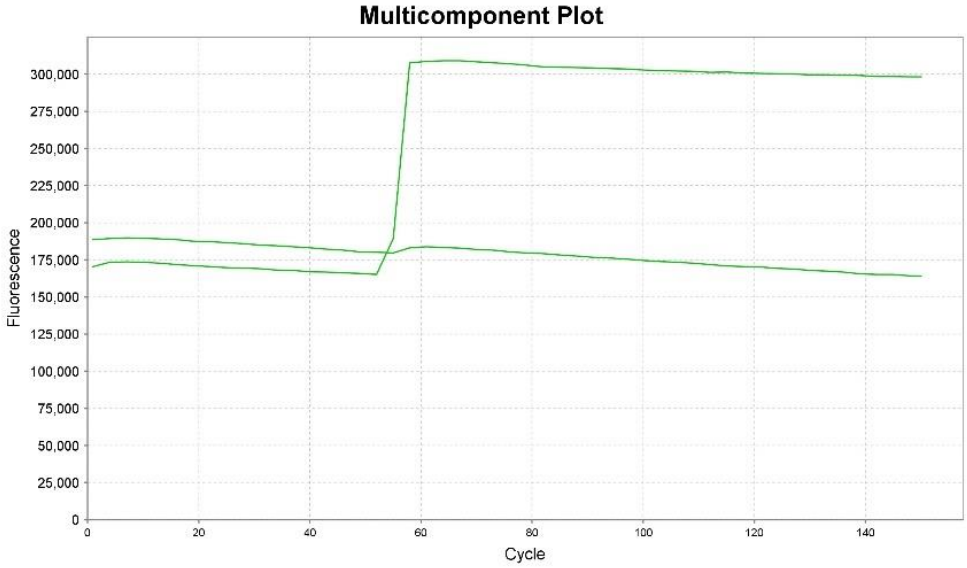

Figure S2. The fluorescence intensity changes when the concentration ratio of ABD is 1:1:1.
